# Upregulation of Nrf2 attenuates* Angiostrongylus cantonensis*-induced parasitic meningitis in mice

**DOI:** 10.1186/s13071-025-06724-z

**Published:** 2025-04-03

**Authors:** Chii-Wen Chou, Chia-Chun Huang, Ke-Min Chen, Chun-I Wang, Wan-Jing Chen, Chiung-Hung Hsu, Shih-Chan Lai, Shyun Chou, Yu-Kang Chang, Kuan-Yu Lin, Chih-Hao Chiu, Cheng-You Lu

**Affiliations:** 1 Division of Neurosurgery, Tungs’ Taichung Metroharbor Hospital, Taichung, Taiwan; 2 Department of Emergency Medicine, Kuang Tien General Hospital, Taichung, Taiwan; 3 Department of Parasitology, Chung Shan Medical University, Taichung, Taiwan; 4 Department of Biochemistry, School of Medicine, China Medical University, Taichung, Taiwan; 5 Medical Research Center, Cardinal Tien Hospital, New Taipei City, Taiwan; 6 Department of Pathology and Laboratory Medicine, Taichung Veterans General Hospital, Taichung, Taiwan; 7 Department of Veterinary Medicine, College of Veterinary Medicine, National Chung Hsing University, Taichung, Taiwan; 8 Department of Medical Research, Tungs’ Taichung MetroHarbor Hospital, Taichung, Taiwan; 9 Department of Nursing, Jenteh Junior College of Medicine, Nursing and Management, Miaoli, Taiwan; 10 Department of Nursing, Central Taiwan University of Science and Technology, Taichung, Taiwan; 11 Department of Post-Baccalaureate Medicine, College of Medicine, National Chung Hsing University, Taichung, Taiwan

**Keywords:** *A. cantonensis*, Anti-inflammation, Antioxidant, Nrf2, Parasitic meningitis

## Abstract

**Background:**

*Angiostrongylus cantonensis* is a food-borne parasite that can infect mammals, including humans, causing angiostrongyliasis. The nuclear factor E2-related factor 2 (Nrf2) is a transcription factor that plays a crucial role in the host’s antioxidant defense and inflammation mechanisms. Herein, this study investigates the anti-inflammatory effects of Nrf2 in *A. cantonensis*-induced parasitic meningitis in mice.

**Methods:**

We used animal infection and treatment, larvae collection, western blotting, enzyme-linked immunosorbent assay (ELISA), hematoxylin and eosin (H&E) stain, blood–brain barrier (BBB) permeability assays, and an NAD(P)H quinone dehydrogenase 1 (NQO1) enzyme activity, reactive oxygen species (ROS), and superoxide dismutase (SOD) assay kit in this study.

**Results:**

Our findings revealed that larvae recovery, BBB permeability, and inflammatory mediators (interleukin (IL)-1β, IL-6, IL-17A, and tumor necrosis factor (TNF)-α) were increased in *A. cantonensis*-infected mice. However, p-Nrf2 levels were slightly increased in infected groups. To better understand the modulatory role of Nrf2 in the parasitic meningitis, we also treated *A. cantonensis*-infected mice with oltipraz (an Nrf2 activator) and trigonelline (an Nrf2 inhibitor). The larvae recovery, BBB permeability, and levels of inflammatory mediators were significantly decreased in the albendazole alone, oltipraz, and albendazole–oltipraz co-treatment groups, particularly in albendazole–oltipraz co-treatment groups. In contrast, trigonelline treatment resulted in increased levels of larvae recovery, BBB permeability, and inflammatory mediators. Moreover, since Nrf2 is involved in the regulation of antioxidant enzymes, we also examined the expression of ROS, NQO1, and SOD. ROS levels were significantly increased in infected groups but decreased in the albendazole alone, oltipraz alone, and albendazole–oltipraz co-treatment groups. NQO1 and SOD levels were significantly decreased in infected groups, but these levels were notably restored during treatment with albendazole alone, oltipraz alone, and albendazole–oltipraz co-treatment.

**Conclusions:**

Our findings revealed the albendazole–Nrf2 activator co-treatment effectively suppressed excessive inflammation compared with the anthelmintics drug (albendazole) treatment alone, and Nrf2 activation might produce a synergistic effect in the inflammatory response of the brain in mice with angiostrongyliasis.

**Graphical Abstract:**

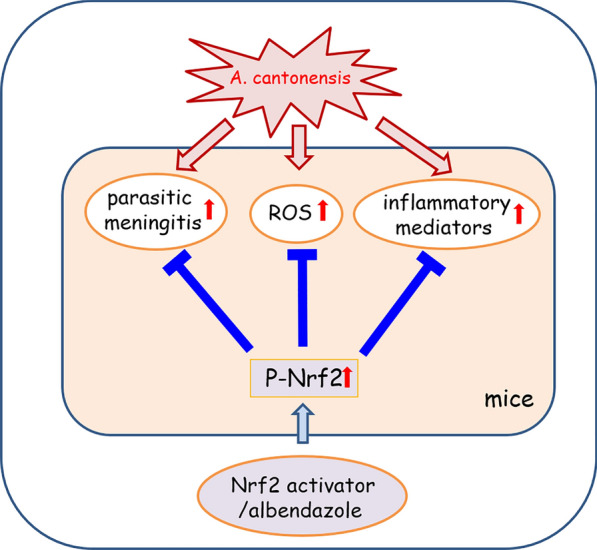

## Background

*Angiostrongylus cantonensis* is a foodborne parasite and zoonotic nematode. Besides other mammals, human and mice are nonpermissive hosts and can develop angiostrongyliasis when they unintentionally consume raw or undercooked food items, such as vegetables, snails, or slugs containing third-stage larvae (L3) [1–3]. This infection can lead to severe central nervous system (CNS) inflammation, demyelination, eosinophilic meningitis, or eosinophilic meningoencephalitis [2, 4, 5]. Parasitic infections often lead to the upregulation of many inflammatory mediators, including Th1 cytokines (interleukin (IL)-1β), Th2 cytokines (IL-5, IL-6 or IL-10), Th17 cytokines (IL-17), cyclooxygenase-2 (COX-2), tumor necrosis factor-α (TNF-α), and nuclear factor (NF)-κB [6–11]. These mediators are typically expressed at low levels underlying normal physiological conditions but are highly induced during inflammation or pathological processes. Moreover, an increasing number of studies have revealed that TNF-α, IL-1β, and IL-17 [7, 8, 11] may participate in the pathogenesis of CNS inflammation during *A. cantonensis* infection. Thus, suppressing *A. cantonensis*-mediated CNS inflammation, eosinophilic meningitis, eosinophilic meningoencephalitis, and the associated inflammatory mediators are critical.

The activation of nuclear factor E2-related factor 2 (Nrf2) serves as an effective antioxidant defense mechanism utilized by host cells to counteract oxidative stress [12, 13]. Furthermore, upon parasite infection in macrophages, Nrf2 activators improve the outcome of severe malaria infection [14], and Nrf2 induction also reduces inflammation with *Trypanosoma cruzi* [15] and *Leishmania* infection [16]. As noted, Nrf2 plays an important protective role in parasite infection. However, there are few studies of Nrf2 in nematodes, particularly in the context of *A. cantonensis* infection. Additionally, our previous studies have demonstrated that modulation of heme oxygenases (HO-1) activation can attenuates parasitic meningitis during *A. cantonensis* infection [8]. Further, Nrf2 is the upstream of HO-1 pathway and can regulate HO-1 expression [13, 17]. However, little is known about the signaling mechanisms, particularly regarding the protective effect of Nrf2 in *A. cantonensis*-induced parasitic meningitis. With this in mind, this study utilized mice infected with *A. cantonensis* as a model to investigate whether upregulation of Nrf2 could attenuate *A. cantonensis*-induced parasitic meningitis.

## Methods

### Chemicals reagents and antibodies

Phospho-Nrf2 (Ser40) (p-Nrf2) antibody was obtained from Thermo Fisher Scientific (Dreieich, Germany), and β-actin antibody was obtained from Santa Cruz Biotechnology Inc. (CA, USA). Albendazole, an anthelmintic or anti-worm medication, trigonelline (a Nrf2 inhibitor), and oltipraz (a Nrf2 activator) were purchased from Sigma (St. Louis, MO, USA). Albendazole, trigonelline, and oltipraz were dissolved in dimethylsulfoxide (DMSO) and administered to animals at a final concentration of 0.1% DMSO.

### Experimental animal

Five-week-old male BALB/c mice were purchased from the National Laboratory Animal Center (Taipei, Taiwan) and were housed on a 12 h light/ dark cycle with free access to water and food. All surgical procedures and postoperative care were conducted in accordance with the Guide for the Care and Use of Laboratory Animals of Hualien Tzu Chi Hospital.

### Animal infection

The infective larvae (L3) of *A. cantonensis* were obtained from *Achatina fulica* snails purchased from Heping District (Taichung, Taiwan). The L3 larvae were liberated from the minced snail tissues by digestion with 0.6% (w/v) pepsin-HCl (pH 2–3) (Sigma, USA). The identity of the *A. cantonensis* L3 larvae identity was confirmed on the basis of previously described methods [18]. Mice were infected with 30 *A. cantonensis* L3 larvae by oral inoculation and were sacrificed 30 days post-infection (P.I.) as the infected groups. Control mice received only water and were also sacrificed on day 30 P.I. The method of euthanasia in mice was exposure to carbon dioxide (CO_2_) flow for at least 1 min until respiratory arrest. Cervical dislocation was then performed as a confirmatory method of euthanasia prior to necropsy [8].

### Treatment of animals

Total of mice were randomly divided into five treated groups (five mice/ group). Five groups were treated with either 10 mg/kg/day albendazole (stomach intubation), 30 mg/kg/day trigonelline (intraperitoneal), 100 mg/kg/day oltipraz (intraperitoneal), a combination of 10 mg/kg/day albendazole (stomach intubation) and 30 mg/kg/day trigonelline (intraperitoneal), or a combination of 10 mg/kg/day albendazole (stomach intubation) and 100 mg/kg/day oltipraz (intraperitoneal) for 19 consecutive days. Drug administration was initiated on day 6 and continued until day 24 after infection. All mice were sacrificed 30 days P.I. The animal infection model was described and slightly modified on the basis of a previous study [8].

### Worm recovery from brain

Each brain was dissected into small pieces and homogenized separately in 15 ml of phosphate-buffered saline (PBS) (pH 7.4) containing 0.25% sodium citrate, followed by centrifugation (1400*g* for 10 min). Larval counts were assessed under a dissecting microscope at 25× magnification. All visible larvae were counted and the larvae recovery method was slightly modified on the basis of a previous study [8].

### Brain samples collection

Brains are dissected and placed in powdered dry ice for storage at −80 °C. The sections (20 μm) at the level of striatum were cut at −18 °C using a cryostat and collected on the slides coated with VECTABOND® Reagent (Vector Labs), then stored at −80 °C until immunostaining. All brains tissue extracts from each group were obtained by homogenizing 100 mg of tissue per ml in T-PER™ Tissue Protein Extraction Reagent (Thermo Fisher Scientific, Waltham, USA) containing 10 uM sodium, 0.1 mM PMSF, and 20 ug/mL leupeptin on ice. The mixture was then centrifuged at 10,000*g* (4 °C for 30 min), and the homogenizing supernatants were collected and stored at −80 °C for further experiments.

### Western blotting

The western blotting analysis were described and slightly modified on the basis of previous studies [19, 20]. The homogenates protein concentrations were determined by Bradford Assay (Bio-Rad, Hercules, CA), after which the protein samples were separated by 10–12% sodium dodecyl sulfate–polyacrylamide gel electrophoresis. The resolved proteins are then transferred to a polyvinylidene fluoride membrane (Merck Millipore, Massachusetts). The membrane is blocked with 5% skim milk in Tris-buffered saline (TBS) (pH 7.4) and then exposed to the appropriate antibodies. All bands are visualized using horseradish peroxidase-conjugated secondary antibodies (Santa Cruz Biotechnology, California, California) through an enhanced chemiluminescence system (Merck Millipore, Massachusetts).

### Enzyme-linked immunosorbent assay (ELISA) assay

The TNF-α, IL-1β, IL-6, and IL-17A protein levels were measured by TNF-α (ab100785), IL-1β (ab100768), IL-6 (ab100713), and IL-17A (ab199081) ELISA kit (Abcam, MA, USA) following the manufacturer’s instructions. The optical density (O.D.) was tested spectrophotometrically at 450 nm on a Synergy LX Multi-Mode Reader (Biotek; CA, USA). The ELISA analysis as described in a previous study with slightly modified methods [8].

### Histology

Immediately after removal, mouse brains were fixed in 10% neutral buffered formalin for 24 h. The fixed brains underwent dehydration using the 50%, 75%, 95%, and 100% ethanol that was later replaced with xylene. The dehydrated brains were embedded in paraffin at 55 °C for 24 h, and several serial sections (10 μm thick) were cut. The sections were subsequently stained with hematoxylin and eosin (HE) stain (Leica Biosystems, Germany). Pathological changes in the sections were examined using a microscope system (Olympus Microscope CKX53, Tokyo, Japan).

### Assay of blood–brain barrier (BBB) permeability

A 2% solution of Evans blue (100 mg/kg body weight; Sigma, St. Louis, MO, USA) was used to assess the BBB barrier permeability of brains, and the method was as described, though slightly modified, in a previous study [8]. At 2 h before sacrifice, mice were injected into the tail vein with 2% Evans blue in saline. The average concentration of Evans blue in CSF was calculated from measurement of the absorbance at 620 nm on a Synergy LX Multi-Mode Reader (Biotek; CA, USA).

### Detection of NAD(P)H quinone dehydrogenase 1 (NQO1)

NQO1 enzyme activity levels were measured using the NQO1 activity assay kit (ab184867, Abcam, Cambridge, UK) in accordance with the manufacturing protocol. The assay is based on the dicoumarol-sensitive reduction of water-soluble tetrazolium salt (WST-1) in the presence of menadione, utilizing 10 ug of cellular lysate protein in a 96-well plate format. The progress of the reaction was measured at 1-min intervals by measuring absorbance at 450 nm using a Synergy LX Multi-Mode Reader (Biotek; CA, USA) [21].

### Reactive oxygen species (ROS) superoxide detection

ROS levels were measured using the ROS assay kit (MBS2540517, MyBioSource, Vancouver, Canada) in accordance with the manufacturing protocol. ROS level detection was as described, though slightly modified, on the basis of methods in previous studies [13, 22].

### Superoxide dismutase (SOD) detection

Total SOD (containing cytosolic and mitochondrial SOD enzyme) activity was measured by a commercially available superoxide dismutase activity assay kit (ab65354, Abcam, USA) according to the manufacturer’s instruction. The optical absorbance was read on a Synergy LX Multi-Mode Reader (Biotek; CA, USA) at a wavelength of 450 nm [13].

### Statistical analysis

One-way ANOVA was performed using SigmaPlot 10.0 software (Systat Software Inc., San Jose, CA, USA) and GraphPad Prism 8 for statistical analysis in this study (Systat Software Inc., San Jose, CA, USA). Comparisons between two groups were conducted using Student’s *t*-tests.

## Results

### The changes of larvae recovery, Evans blue units, and p-Nrf-2 levels in the brains of mice infected with *A. cantonensis* infection treated with albendazole combined with oltipraz

**Fig. 1:**
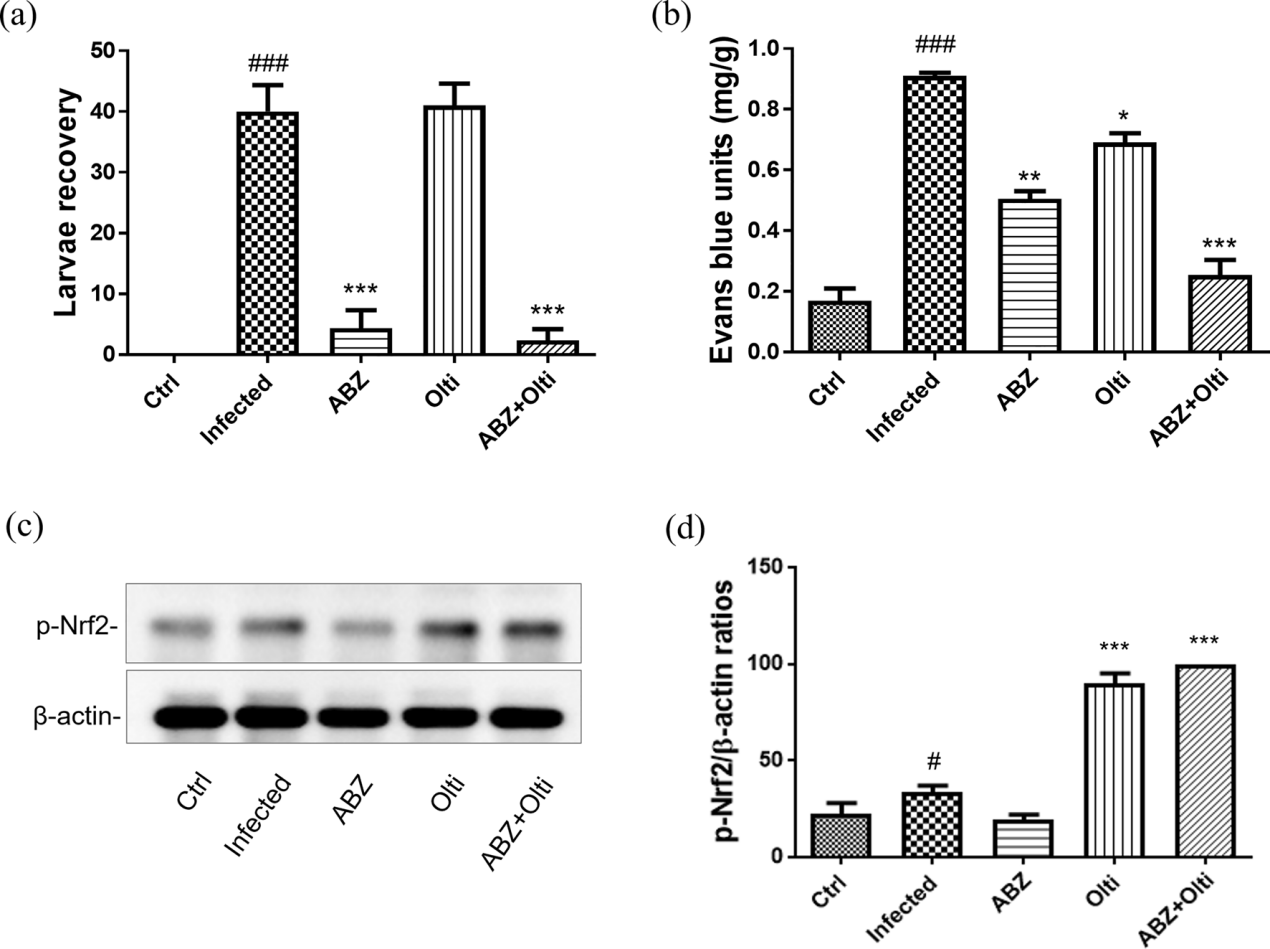
The changes of larvae recovery, Evans blue units, and p-Nrf2 levels in the brains of mice infected with *A. cantonensis*. Larval recovery (**a**), Evans blue units (**b**), and p-Nrf2 (**c**) were detected in all treatment groups. #*P* < 0.05 and ### *P* < 0.001 indicate a significant difference compared with control group. *P < 0.05, ** P < 0.01 and *** P < 0.001 indicate a significant difference compared with infection group. *Ctrl* control group, *Infected* *A. cantonensis* infection group, *ABZ* albendazole treatment group, *Olti* oltipraz treatment group, *ABZ + Olti* albendazole combined with oltipraz treatment group

The larvae recovery and Evans blue units are associated with the development of eosinophilic meningitis, meningoencephalitis and BBB permeability in *A. cantonensis*-infected mice. Thus, we assessed these parameters 30 days P.I. The larvae recovery was significantly increased in the infected groups and oltipraz-only treatment groups compared with control groups and significantly decreased in albendazole-only or albendazole–oltipraz co-treatment groups (Fig. 1a). Additionally, the BBB permeability was assessed by measuring Evans blue concentration in the brains [8]. The results showed that Evans blue units were significantly increased in infected groups compared with the control groups, while they were significantly decreased in the albendazole-only, oltipraz-only, and albendazole–oltipraz co-treatment groups compared with the infected groups, particularly in the albendazole–oltipraz co-treatment groups (Fig. 1b). Furthermore, p-Nrf2 activation plays an important protective role in inflammatory disease. Therefore, we examined p-Nrf2 expression in all groups using Western blotting. Moreover, the p-Nrf2 levels were slightly increased in the infected groups compared with control groups, and significantly increased in the oltipraz-only or albendazole–oltipraz co-treatment groups compared with the infection groups, particularly in albendazole–oltipraz co-treatment groups (Fig. 1c).

### The changes of IL-1β, IL-6, IL-17A, and TNF-α in the brains of mice infected with *A. cantonensis* infection treated with albendazole combined with oltipraz

**Fig. 2:**
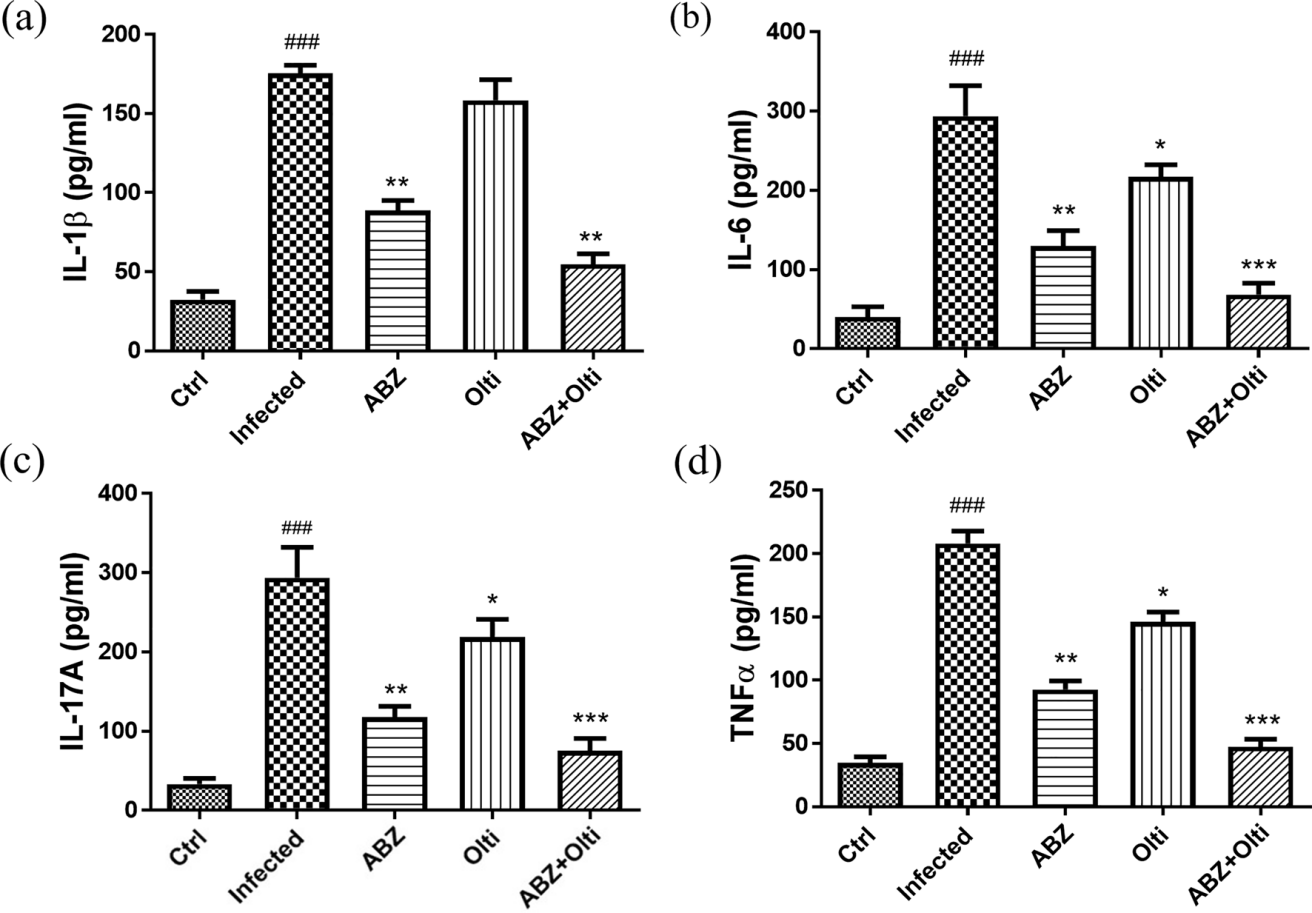
The changes of IL-1β, IL-6, IL-17A, and TNF-α in the brains of mice. IL-1β (**a**), IL-6 (**b**), IL-17A (**c**), and TNF-α (**d**) levels were detected in all treatment groups by ELISA assay. ### *P* < 0.001 indicates a significant difference compared with control group. *P < 0.05, ** P < 0.01 and *** P < 0.001 indicate a significant difference compared with infection group. *Ctrl* control group, *Infected* *A. cantonensis* infection group, *ABZ* albendazole treatment group, *Olti* oltipraz treatment group, *ABZ + Olti* albendazole combined with oltipraz treatment group

The inflammatory mediators IL-1β, IL-6, IL-17A, and TNF-α are associated with the development of inflammation. Therefore, we examined the protein levels of these mediators in all groups using ELISA. As shown in Fig. 2a, b, c and d, IL-1β, IL-6, IL-17A, and TNF-α were significantly increased in the infected groups. In contrast, these mediators were significantly decreased in the albendazole-only, oltipraz-only, or albendazole–oltipraz co-treatment groups compared with the infected groups, particularly in albendazole–oltipraz co-treatment groups.

### The changes of ROS, NQO1 and SOD levels in the brains of mice infected with *A. cantonensis* infection treated with albendazole combining with oltipraz

**Fig. 3:**
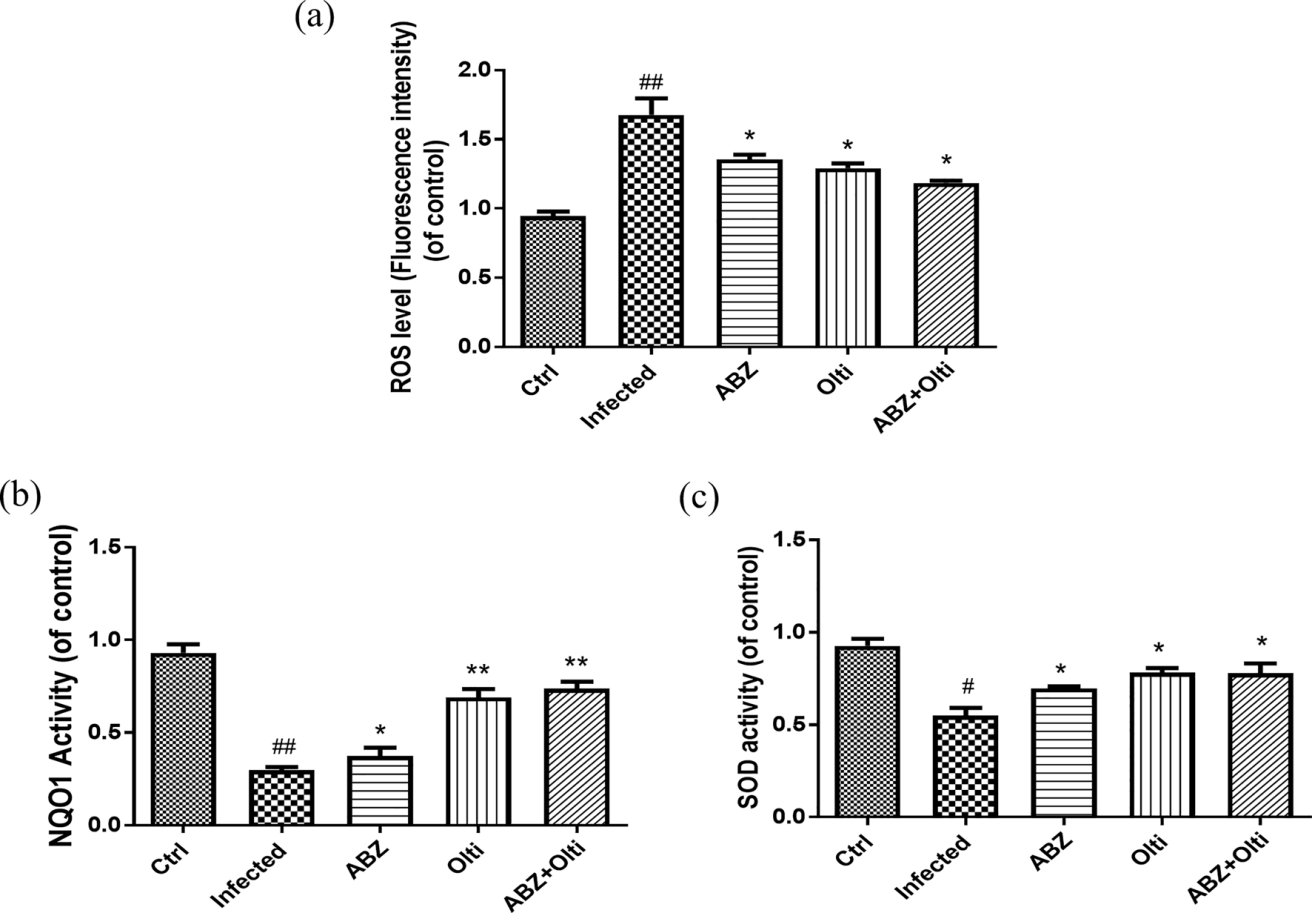
The changes of NQO1, ROS, and SOD levels in the brains of mice. NQO1 (**a**), ROS (**b**), and SOD (**c**) levels were detected in all treatment groups. #*P* < 0.05 and ## *P* < 0.001 indicates a significant difference compared with control group. *P < 0.05, ** P < 0.01 and *** P < 0.001 indicate a significant difference compared with infection group. *Ctrl* control group, *Infected* *A. cantonensis* infection group, *ABZ* albendazole treatment group, *Olti* oltipraz treatment group, *ABZ + Olti* albendazole combined with oltipraz treatment group

The result in Fig. 1 showed that Nrf2 levels were changed in *A. cantonensis* infection and treatment groups. Moreover, Nrf2 plays an antioxidant role in inflammatory diseases. Furthermore, the ROS, NQO1, and SOD are associated with antioxidant defense mechanisms in inflammatory conditions. Therefore, we examined the ROS (Fig. 3a), NQO1 (Fig. 3b) and SOD (Fig. 3c) across all groups. ROS levels were significantly increased in the infected groups and significantly decreased in the albendazole-only, oltipraz-only, and albendazole–oltipraz co-treatment groups compared with the infected groups, particularly in albendazole–oltipraz co-treatment groups. The NQO1 and SOD were significantly decreased in the infected groups and slightly increased in the albendazole-only groups. Nevertheless, NQO1 and SOD were significantly increased in the oltipraz-only or albendazole–oltipraz co-treatment groups compared with the infected groups.

### The changes of larvae recovery, Evans blue units, and p-Nrf-2 levels in the brains of mice infected with *A. cantonensis* infection treated with albendazole combining with trigonelline

**Fig. 4:**
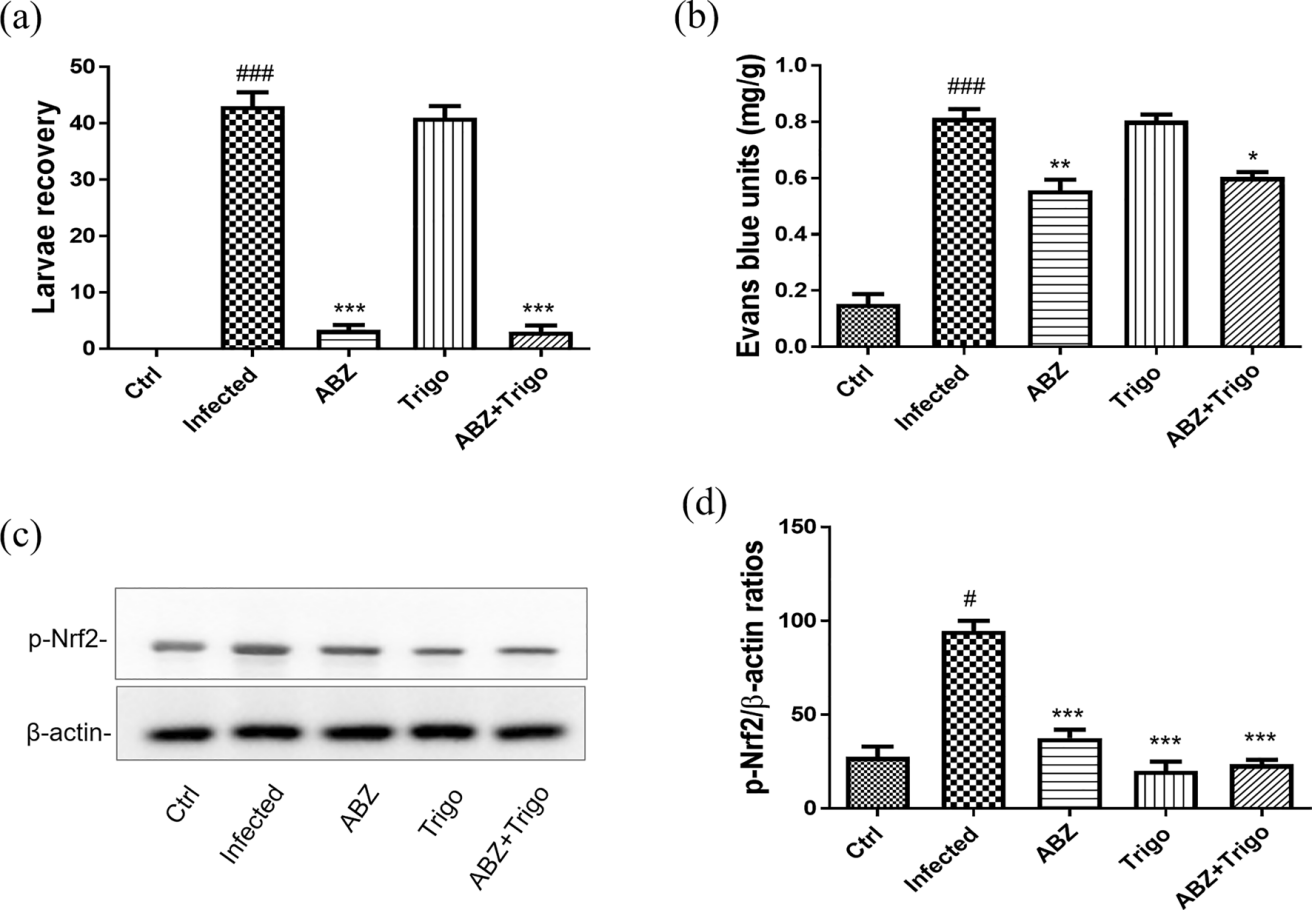
The changes of larvae recovery, Evans blue units and p-Nrf2 levels in the brains of mice infected with *A. cantonensis* infection. Larval recovery (**a**), Evans blue units (**b**), and p-Nrf2 (**c**) were detected in all treatment groups. #*P* < 0.05 and ### *P* < 0.001 indicate a significant difference compared with control group. *P < 0.05, ** P < 0.01 and *** P < 0.001 indicate a significant difference compared with infection group. *Ctrl* control group, *Infected* *A. cantonensis* infection group, *ABZ* albendazole treatment group, *Trigo* trigonelline treatment group, *ABZ + Trigo* albendazole combined with trigonelline treatment group

To reconfirm the protective effects of Nrf2, we conducted experiments to block Nrf2 activation using trigonelline, a potent inhibitor of Nrf2 [23, 24]. The recovery of larvae was significantly increased in the infected groups and trigonelline-only treatment groups compared with the control groups and significantly decreased in albendazole-only or albendazole–trigonelline co-treatment groups (Fig. 4a). Additionally, the Evans blue units were significantly increased in the infected groups compared with the control groups and significantly decreased in the albendazole-only. However, the Evans blue units of albendazole–trigonelline co-treatment groups showed a slightly increased compared with albendazole-only treatment (Fig. 4b). Moreover, the p-Nrf2 levels were slightly increased in the infected groups compared with the control groups, but significantly decreased in the trigonelline-only or albendazole–trigonelline co-treatment groups compared with the infected groups (Fig. 4c).

### The changes of IL-1β, IL-6, IL-17A, and TNF-α in the brains of mice infected with *A. cantonensis* infection treated with albendazole combined with trigonelline

**Fig. 5:**
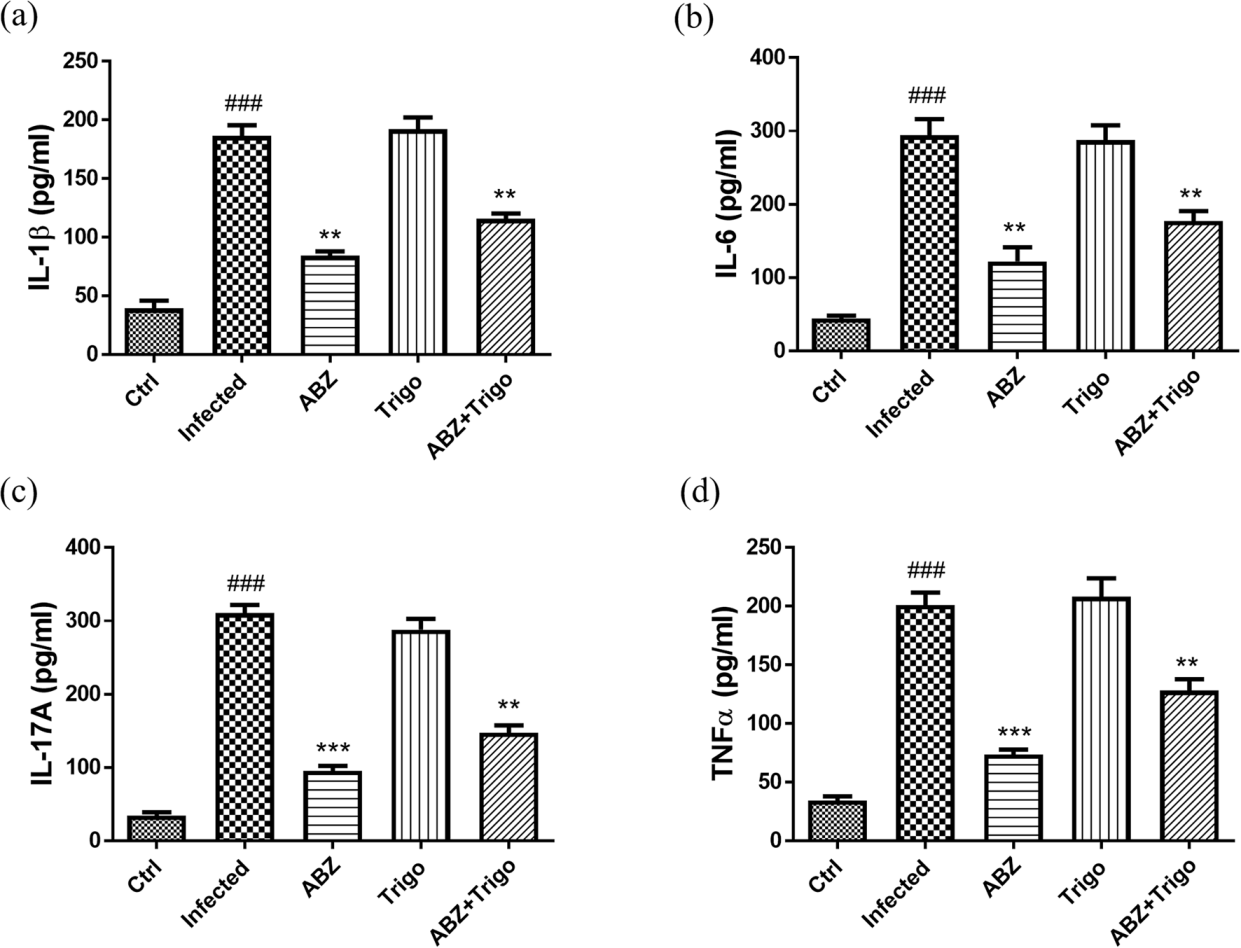
The changes of IL-1β, IL-6, IL-17A, and TNF-α in the brains of mice. IL-1β (**a**), IL-6 (**b**), IL-17A (**c**), and TNF-α (**d**) levels were detected in all treatment groups by ELISA assay. ### *P* < 0.001 indicates a significant difference compared with control group. ** P < 0.01 and *** P < 0.001 indicate a significant difference compared with infection group. *Ctrl* control group, *Infected* *A. cantonensis* infection group, *ABZ* albendazole treatment group, *Trigo* trigonelline treatment group, *ABZ + Trigo* albendazole combined with trigonelline treatment group

The ELISA assay was used to examine the changes of the inflammatory mediators IL-1β, IL-6, IL-17A, and TNF-α during trigonelline treatment. IL-1β (Fig. 5a), IL-6 (Fig. 5b), IL-17A (Fig. 5c), and TNF-α (Fig. 5d) were significantly increased in the infected groups and trigonelline-only treatment groups. Moreover, IL-1β, IL-6, IL-17A, and TNF-α were significantly decreased in the albendazole-only and albendazole–trigonelline co-treatment groups compared with the infected groups. However, the levels of these inflammatory mediators in the albendazole–trigonelline co-treatment groups were slightly higher compared with those in the albendazole-only treatment groups.

### The changes of ROS, NQO1, and SOD in the brains of mice infected with *A. cantonensis* infection treated with albendazole combining with trigonelline

**Fig. 6:**
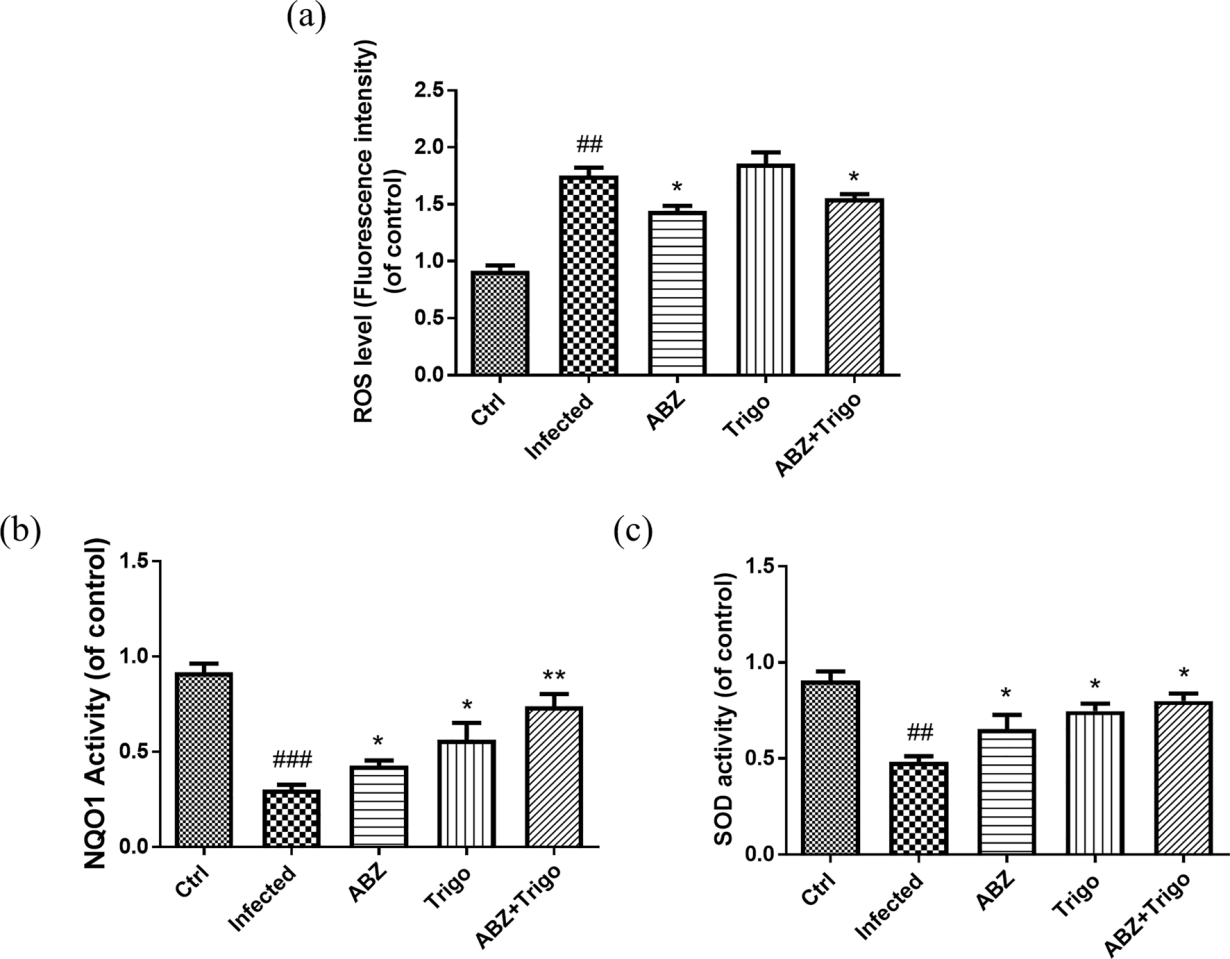
The changes of NQO1, ROS and SOD levels in the brains of mice. NQO1 (**a**), ROS (**b**), and SOD (**c**) levels were detected in all treatment groups. ## *P* < 0.005 and ###* P* < 0.001 indicate a significant difference compared with control group. * P < 0.05 and ** P < 0.01 indicate a significant difference compared with infection group. *Ctrl* control group, *Infected* *A. cantonensis* infection group, *ABZ* albendazole treatment group, *ABZ + Olti* albendazole combined with oltipraz treatment group, *ABZ + Trigo* albendazole combined with trigonelline treatment group

The ROS (Fig. 6a) levels were significantly increased in the infected groups, but significantly decreased in the albendazole-only treatment groups. During trigonelline-only treatment, trigonelline had no effect on ROS levels. However, in albendazole–trigonelline co-treatment groups, the ROS levels were slightly higher than albendazole-only treatment groups. The levels of NQO1 (Fig. 6b) and SOD (Fig. 6c) were significantly decreased in the infected groups, albendazole-only, trigonelline-only, and albendazole–trigonelline co-treatment groups. However, during albendazole-only treatment, NQO1 and SOD levels were slightly increased compared with the infected groups.

### Histopathological examinations on the brains of mice infected with *A. cantonensis* infection treated with albendazole combining with oltipraz or trigonelline

**Fig. 7:**
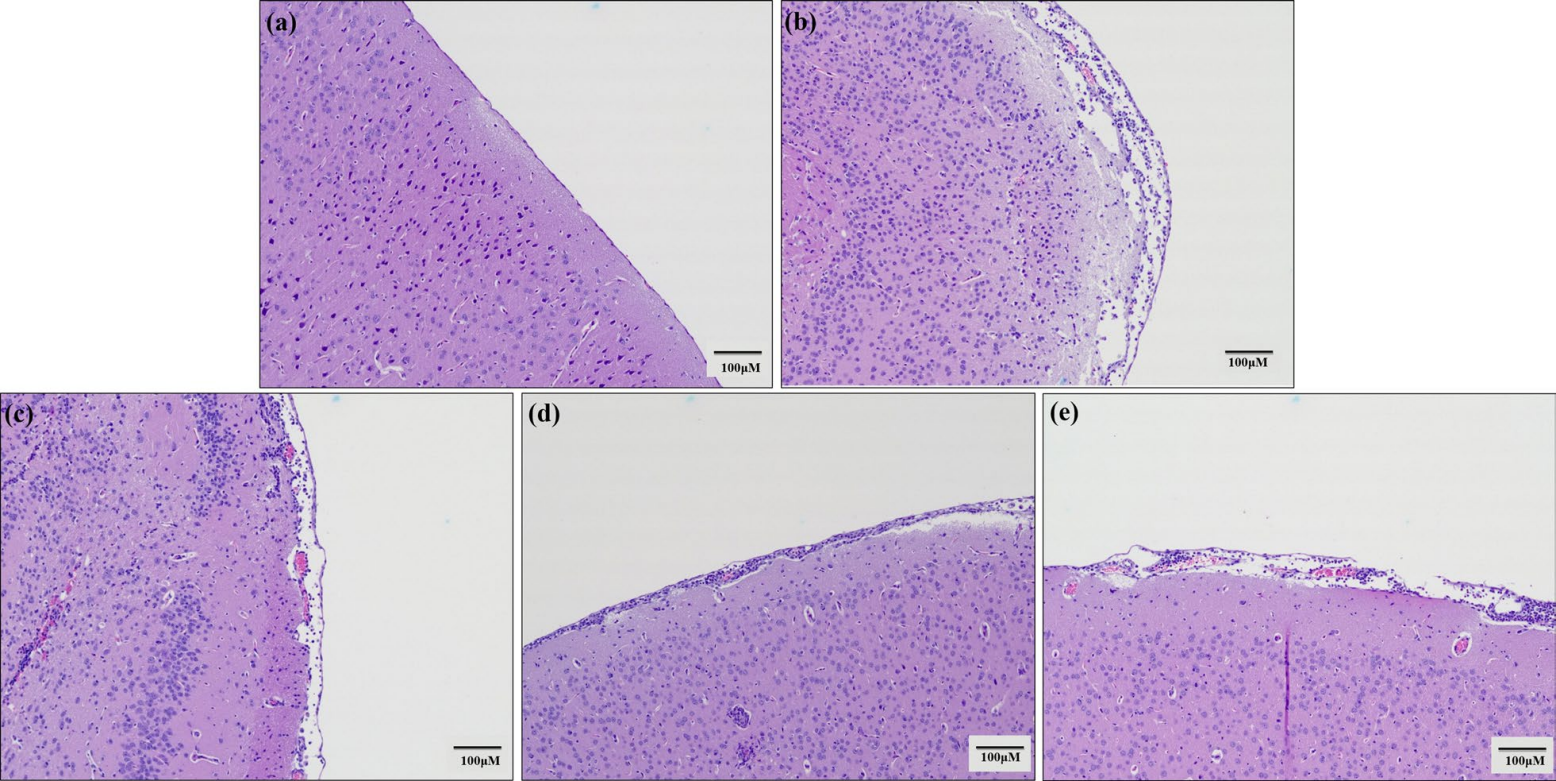
Histopathological examinations from the brain of mice. (**a**) Control group (*Ctrl*). (**b**) *Angiostrongylus cantonensis* infection group (*Infected*). (**c**) Albendazole treatment group (*ABZ*). (**d**) Albendazole combined with oltipraz treatment group (*ABZ + Olti*). (**e**) Albendazole combined with trigonelline treatment group (*ABZ + Trigo*)

Pathological changes in the brain sections of the mice were stained using HE stain and examined under a microscope system (Olympus Microscope CKX53, Tokyo, Japan). In *A. cantonensis*-infected mouse groups, the brain tissue showed severe hemorrhage, as well as enlarged spaces and leukocyte infiltration into the subarachnoid space compared with the control groups. In the albendazole-only treatment groups, the hemorrhage and space enlargement were moderately reduced, although a slight leukocyte infiltration remained. In contrast, the hemorrhage, space enlargement, and leukocyte infiltration were significantly decreased in albendazole–oltipraz co-treatment groups compared with the *A. cantonensis*-infected groups. However, the albendazole–trigonelline co-treatment groups reversed the beneficial effects of albendazole (Fig. 7).

## Discussion

Anthelmintics, such as albendazole, are the most important drugs for treatment of angiostrongyliasis [25, 26]. Additionally, many studies reported that albendazole–steroid or albendazole–anti-inflammatory drug co-therapy might produce a synergistic effect, slowing the development and pathological process of angiostrongyliasis [8, 27–30]. Previous studies revealed that modulation of HO-1 and inflammatory mediator activation can attenuate parasitic meningitis during *A. cantonensis* infection [8, 10]. Additionally, various studies have demonstrated that Nrf2 is the upstream of HO-1 pathway and can regulate both HO-1 and inflammatory mediator activation [13, 17]. In this study, we utilized Nrf2 activator oltipraz and Nrf2 inhibitor trigonelline to comprehensively investigate the role of Nrf2 and its association with the inflammatory mediator pathway in angiostrongyliasis of *A. cantonensis*-infected mice.

Nrf2 offers several advantages for tissue protection and regulates the expression and coordinated induction of a range of genes that encode detoxifying enzymes, antiapoptotic proteins, and proteasomes [13, 31, 32]. Although Nrf2 is normally expressed at low levels in most organs, it can be highly inducible in response to various stimuli, providing cell protection against inflammatory reaction-capability associated with the resolution of inflammation [12, 13, 20]. However, while Nrf2 levels may vary in response to certain stimuli or inflammatory responses, this phenomenon does not always suffice to suppress inflammation, such as in the H_2_O_2_-induced astrocyte injury [13], IL-17A-induced inflammatory response of synoviocytes [20], LPS-stimulated macrophage-mediated inflammation [33], and infections caused by *Trypanosoma cruzi* [15, 34], malaria [34–36], and *Leishmania* [16, 34]. Therefore, the Nrf2 pathway may be targeted for the prevention and treatment of parasitic infection [16, 34, 36].

In the present study, *A. cantonensis*-induced parasitic meningitis was found to upregulate Nrf2 expression compared with the control group; however, the increased levels of Nrf2 were insufficient to reduce the inflammation of brain injury. Therefore, we aimed to explore whether inducing Nrf2 activation could mitigate the inflammation of angiostrongyliasis. Moreover, previous reports indicate that Nrf2 activators ameliorate the inflammatory response associated with malaria infection [34, 36], and Nrf2 induction has been shown to improve outcomes in *Trypanosoma cruzi* infection [15, 34]. Additionally, the Nrf2 pathway may be targeted to prevent toxoplasmosis [37] and Vatankhah et al. reported that Nrf2 pathway is one of the antioxidant mechanisms that combat oxidative stress during parasites infection [34]. Therefore, we first used oltipraz, a Nrf2 activator, to confirm whether the Nrf2 activation could improve *A. cantonensis*-mediated CNS inflammation. Our results showed that BBB permeability, worm recovery, and inflammatory mediators were enhanced in mice with eosinophilic meningitis or meningoencephalitis caused by *A. cantonensis*-infection. The levels of these measures were significantly reduced in the albendazole, oltipraz, and albendazole–oltipraz co-treatment groups compared with the *A. cantonensis*-infected mice. This reduction was most pronounced in the albendazole–oltipraz co-treatment group. In contrast, p-Nrf2 levels showed a slight increase following treatment with albendazole, oltipraz, and their combination. Notably, the albendazole–oltipraz co-treatment group exhibited a significant elevation in p-Nrf2 levels, suggesting that Nrf2 activation may play a crucial anti-inflammatory role in mitigating *A. cantonensis*-induced inflammation. These findings suggest that Nrf2 activation could potentially reduce the progression of *A. cantonensis*-induced inflammation. To better evaluate the role of Nrf2, we treated *A. cantonensis*-infected mice with trigonelline, an Nrf2 inhibitor. Our results showed that both trigonelline treatment alone and the combination of trigonelline with albendazole reversed reduction of *A. cantonensis*-induced inflammation in mice following treatment with albendazole. These results indicate that modulation of Nrf2 activation protects against inflammation in *A. cantonensis*-infected mice.

In the present study, we found that angiostrongyliasis, eosinophilic meningitis, and BBB damage were markedly reduced following treatment with oltipraz, a Nrf2 activator. Additionally, Nrf2 plays an antioxidant role in inflammatory diseases, and the levels of ROS, NQO1 and SOD are associated with antioxidant defensive mechanisms in these conditions (ROS levels were increased, while NQO1 and SOD levels were decreased) [13, 38, 39]. Furthermore, the induction of SOD and NQO1 induction can combat oxidative stress-induced cellular damage, and the upregulation of these two proteins may be regulated by Nrf2 activation [39–41]. Therefore, we also examined the changes to ROS, NQO1, and SOD levels in this study. ROS levels were increased following the challenge with *A. cantonensis*, whereas subsequent treatment with oltipraz, a Nrf2 activator, attenuated ROS levels and the production of inflammatory mediators (TNF-α, IL-1β, IL-6, and IL-17A). In contrast, treatment with trigonelline, a Nrf2 inhibitor, exacerbated angiostrongyliasis and increased inflammatory mediators. Notably, NQO1 and SOD levels showed an opposite trend to ROS levels in this study. These results provide new insights into the evaluation and mechanisms of the Nrf2 action, supporting the hypothesis that Nrf2 activation might contribute to reduce the progression of *A. cantonensis*-induced inflammation. Moreover, a better understanding of the signal transduction mechanisms underlying antioxidant (neuroprotective) regulation will create opportunities for developing anti-inflammatory therapeutic strategies. However, the present study has some limitations. First, Nrf2 function is associated with Kelch-like ECH-associated protein 1 (Keap1) and antioxidant response elements (ARE). Further studies are required to clarify the roles of Keap1 and ARE in *A. cantonensis*-induced inflammation. Second, this study only utilized Nrf2 inhibitor and Nrf2 activator in an in vivo design. Additional studies are needed to verify these results and support potential applications in an in vitro model, as well as to use Nrf2 knockout mice to examine and confirm the anti-inflammatory effects of Nrf2 in *A. cantonensis* infection. Finally, further research is needed to support potential clinical applications.

## Conclusions

We found that *A. cantonensis* induced parasitic meningitis, induced inflammation, and slightly increased of p-Nrf2 in the brains of mice. In contrast, the albendazole–Nrf2 activator co-treatment effectively suppressed excessive inflammation compared with albendazole treatment alone. Ultimately, we found that upregulate the Nrf2 levels could attenuate *A. cantonensis*-induced parasitic meningitis.

## Data Availability

No datasets were generated or analyzed during the current study.

## Abbreviations

BBB: Blood–brain barrier
IL: Interleukin
TNF: Tumor necrosis factor
ROS: Reactive oxygen species
NQO1: NAD(P)H quinone dehydrogenase 1
SOD: Superoxide dismutase
COX-2: Cyclooxygenase-2
Nrf2: Nuclear factor E2-related factor 2
HO-1: Heme oxygenase-1
DMSO: Dimethylsulfoxide
P.I.: Post-infection
PBS: Phosphate-buffered saline
ELISA: Enzyme-linked immunosorbent assay
O.D.: Optical density
Keap1: Kelch-like ECH-associated protein 1
ARE: Antioxidant response elements
